# Website Visitors Asking Questions Online to Lung Cancer Specialists: What Do They Want To Know?

**DOI:** 10.2196/ijmr.1749

**Published:** 2013-08-06

**Authors:** Romane M Schook, Cilia Linssen, Jan Festen, Franz MNH Schramel, Ernst Lammers, Peter Zaanen, Pieter E Postmus

**Affiliations:** ^1^VU University Medical CenterDepartment of Pulmonary DiseasesAmsterdamNetherlands; ^2^Lung Cancer Information CenterMaarsbergenNetherlands; ^3^St Antonius Hospital NieuwegeinNieuwegeinNetherlands; ^4^Gelre ZiekenhuizenZutphenNetherlands; ^5^Mezanet Interactive Media AmsterdamAmsterdamNetherlands

**Keywords:** Internet, lung neoplasms, medical informatics, information services, patient education, information needs, caregivers

## Abstract

**Background:**

In 2003 the Dutch Lung Cancer Information Centre (Longkanker Informatie Centrum) launched a website containing information on lung cancer accessible to anyone.

**Objective:**

Our study aim was to inventorize the information needs of the visitors of this website by analyzing the questions they asked the lung cancer specialists in the websites interactive section “Ask the Physician”.

**Methods:**

The first 2000 questions posted up until May 2006 have been classified by visitors’ wish, type of required information, identity, gender, and phase during treatment course.

**Results:**

Our results show that 1893 (1158/1893, 61%) of the questions were asked by a loved one/caregiver and (239/1893 13%) by patients. 1 out of 3 questions was asked by a daughter/grand-daughter. Most questions concerned specific information on lung cancer and lung cancer course (817/1893, 43%). The most inquired specific information topics were therapy side effects, diagnostics, general information on lung cancer, and regular therapy. Furthermore, questioners wanted to verify their own doctor’s information (122/1893, 6%), a diagnosis (267/1893, 14%), and a prognosis (204/1893, 11%).

**Conclusions:**

Lung cancer patients and their caregivers asked the most questions in the interactive website section. The most frequently requested information was more detailed information. These include specific information on lung cancer (regular therapy, diagnostics, and disease symptoms), verification of what the doctor has said, diagnosis, and prognosis. Most of the requested information could have been obtained from treating specialists, indicating that current information supply to lung cancer patients and their caregivers may not be matching their needs sufficiently. The further implementation of an online dialogue with lung cancer specialists might be a solution.

## Introduction

The Internet has changed the position of patients within the healthcare system. Currently, the Internet is widely used as a resource for health related information [1-4]. Ybarra et al [5] have reported a percentage of 73% Internet use among Americans, of whom 56% reported using the Internet as a resource for health information. A few health care providers already utilize the potential of the Internet [6-9] such as, the “emaildoctor” [10]. However, these physicians are still forerunners and not disease specific specialists, possibly making information superficial and not up-to-date, resulting in resistance against these practices among medical specialists.

As a result of an initiative of doctors, patients, nurses, and other professionals involved with lung cancer, the Dutch Lung Cancer Information Centre (DLIC) was founded. There were not a lot of information available on lung cancer in the Netherlands and lung cancer patient groups were poorly organised [11].

This centre is meant for lung cancer patients, their relatives or loved ones, and people seeking information about lung cancer. The centre of the activities of DLIC is the website [12]. Since its launch in 2003, the DLIC website has been visited very often and has reached a steady number of 20,000 visitors per month. The number of monthly visitors are striking, considering that lung cancer incidence and prevalence respectively are around 10,500 and 14,000 per year in the Netherlands [13,14]. Results from our previous study have shown that caregivers of lung cancer patients are the largest group of visitors of the website [11]. Deducted from the total number of visitors and visitor type [11], around 1600 patients and 11,800 caregivers visit the DLIC website each month [13].

The most popular page of the DLIC website is the interactive section “Ask the Physician”, which was launched in March 2004 [15]. Through this web page, visitors can ask lung cancer specialists specific questions about lung cancer. Since the launch of this interactive web page 7 years ago, approximately 6400 questions have been posted. Furthermore, around 500 people per day visit the section “Ask the Physician” to read these questions and their answers.

The large number of questions in the section “Ask the physician” indicates that website questioners, presumably lung cancer patients and their caregivers, are in need of information on lung cancer. Studying these questions might give more insight into the identity of these specific visitors and in their information needs. It is important to define these needs as it might help defining guidelines for a better way of addressing lung cancer information by treating specialists. There are many studies published about looking for health related information on the Internet, but we did not find any studies addressing online interaction between questioners and lung cancer specialists. The aim of this study was to classify the asked questions posed on the DLIC website into categories so as to give an overview of the types of persons who visit the website and their information needs.

## Methods

### Overview

The main objective of the DLIC for answering questions in the interactive section “Ask the Physician” is to give support to questioners, clarify, and indicate where possibilities can be found with their own specialist. If lifestyle advices or smoking were mentioned by questioners, smoking was systematically discouraged, while exercise and a healthy diet were encouraged. Diagnoses were never stipulated, initial opinions were not challenged, and no other treatment suggestions were made.

Every time new visitors used the interactive section to ask a question, they had to fill in a form and give their name and email address. Each form and each question with the matching answer have been carefully read retrospectively by our team members (RMS and CL). After reading, categories were deducted from the form/question/answer according to their content and set in a database. If it was impossible to determine any of the categories of the visitors, items were classified as unknown.

The questions have been categorized into the next items determined by our research group (see Table 1).

### Analysis

The first 2000 questions asked until May 2006 on the webpage “Ask the Physician” [12] have been imported to a Microsoft Access database and then categorized and analysed according above mentioned items.

### Ethical Approval

According to Dutch law, this study does not need approval by an ethical review board.

**Table 1:** Categories.

Categories	Possible outcomes
**Questioner identity**	
	Student
	Patient
	Caregiver: child/grandchild, partner, other family members, no family
	Person who fears lung cancer
	Other
	Unknown
**Gender**	
	Male
	Female
**Phase of illness/phase in lung cancer procedure**	
	Before diagnosis-symptoms only
	Before diagnosis-after X-ray
	After diagnosis
	Time of choosing therapy
	After surgery
	During therapy
	After therapy
	After healing or recovery
	Terminal stage
	After death
	Other
	Unknown
**Type of information requested**	
	Specific information on lung cancer or lung cancer therapy Diagnosis
	Prognosis
	Treatment advice
	Explanation of doctor’s words
	Terminology questions
	Help with a choice
	Lifestyle advice
	Help with essay/paper
	Other
	Unknown
**Specific information: specific information topics**	
	Lung cancer information in general
	Therapy side effects
	Symptoms of disease
	Regular therapy
	Alternative therapy
	Experimental therapy
	Diagnostics
	Lung cancer prevention
	Disease progression
	Other

## Results

### General

Since its launch in March 2004, the webpage “Ask the Physician” has been widely visited. Data on the numbers of visitors, page views, questioners, questions, lung cancer incidence, and prevalence in the Netherlands are not shown in current manuscript but are available on request.

During our defined study period (March 2004-May 2006), 2000 questions have been asked by 1200 people. One person asked 107 questions on her own, and the information seeking behavior of this person was not likely to be representative for the majority of questioners using the interactive webpage. This person was excluded.

Eighty percent (1199/1893, 80%) of the people who asked questions on the interactive webpage asked one question. The rest (694/1893, 20%) asked one or more additional questions. Around 1% of the people asked more than 10 questions. In total 1893 questions have been analysed.

### Who Asks Questions?

Tables 2 and 3 give the demographics of the persons asking questions on the webpage. The majority of questions were asked by caregivers of lung cancer patients (1158/1893, 61%). Thirteen percent (243/1893, 13%) of all the questions were asked by patients. Of the total study group around one third (849/1893, 33%) of questions were asked by daughters and granddaughters. The category “*unknown*” has been applied when demographics of the questioners could not be found.

Regarding the percentages of questions asked by caregivers and lung cancer patients, caregivers asked 4.8 times more questions than patients in a period of 27 months. This means that 212 questions were asked by caregivers per 100 patients per year.

### Moment of Asking Questions

All questions asked by patients and caregivers (n=1394) were asked at different phases during lung cancer procedure. Most questions arose during therapy (376/1394, 27%), after therapy (223/1394, 16%) and after diagnosis (209/1394, 15%). Questions were also asked at the terminal stage of illness (125/1394, 9%), before diagnosis after the first X-ray (112/1394, 8%), and after surgery (98/1394, 7%).

### What Did the Visitors Ask?

Table 4 provides an overview of the wanted information by questioners. Patients (n=243) requested specific information (122/243, 50%), wanted to verify doctor’s information (25/243, 10%) and a diagnosis (20/243, 8%) or a prognosis (19/243, 8%) in the most cases. Other questioners (n=1650) wanted specific information (695/1650, 42%), a diagnosis (247/1650, 15%), a prognosis (185/1650, 11%) and to verify doctor’s information (6%).

The category “verify doctor’s information” means that a questioner checked whether the information given by the specialist was true: “the doctor has told me that I can choose between chemotherapy and radiotherapy as therapy, is this true?” The category “clarify doctor’s explanation” means that the questioner wanted an explanation of what the specialist had said: “My father has lung cancer and will be treated with chemotherapy. The doctor has said that with treatment my father has 30% chance. What does he mean?” The category “unknown” has been applied when the purpose of the questions was unclear or unknown.

### Specific Information Topics on Lung Cancer and Lung Cancer Therapy

In the case of questions regarding specific information, the number of topics asked exceeds the number of requests for information on lung cancer and lung cancer therapy (Tables 4 and 5) because questions generally contained several topics people wanted to know about.

When patients wanted specific information, the most frequently discussed topics were (see Table 5) therapy side-effects (29/145, 20%), diagnostics (28/145, 19%), regular therapy (26/145, 18%), experimental therapy (15/145, 10%) and disease symptoms (14/145, 10%).

When other questioners requested specific information, the most frequently asked questions were about therapy (196/931, 21%), general information on lung cancer (140/931, 15%), diagnostics (113/931, 12%), disease symptoms (109/931, 12%), therapy side effects (100/931, 11%) and disease course (102/931, 11%).

**Table 2:** Questioner’s identity type.

Questioner identity (n questions=1893)	n	%
Caregiver	1158	61.17
Patient	243	12.84
Person who fears lung cancer	239	12.63
Student	55	2.91
Other	30	1.58
Unknown	168	8.87

**Table 3:** Questioner’s identity type by gender.

Category		n	%
**Gender (n questions=1893)**			
	male	415	21.92
	female	1225	64.71
	unknown	253	13.37
**Gender of patients (n=243)**			
	male	66	27.2
	female	144	59.3
	unknown	33	13.6
**Gender of caregivers: male, female, unknown (n=1158)**			
	male	190	16.41
	female	835	72.11
	unknown	133	11.49
**Children/grandchildren (n=849)**			
	male	123	14.49
	female	622	73.26
	unknown	104	12.25
**Partner (n=180)**			
	male	48	26.67
	female	126	70.00
	unknown	6	3.33
**Other family members (n=83)**			
	male	11	13.25
	female	55	66.27
	unknown	17	20.48
**No family (n=46)**			
	male	8	17.39
	female	32	69.57
	unknown	6	13.04

**Table 4:** What was asked in the first instance: topics, patients, and other questioners.

Topics	Patients (n=243) n (%)	Other questioners (n=1650) n (%)	Total group (n=1893) n (%)
Specific information	122 (50.2)	695 (42.12)	817 (43.16)
Verify doctor’s information	25 (10.3)	97 (5.88)	122 (6.44)
Diagnosis	20 (8.2)	247 (14.97)	267 (14.10)
Prognosis	19 (7.8)	185 (11.21)	204 (10.78)
Treatment advice	14 (5.8)	85 (5.15)	99 (5.23)
Other	11 (4.5)	69 (4.18)	80 (4.23)
Terminology	10 (4.1)	66 (4.00)	76 (4.01)
Clarify doctor’s explanation	7 (2.9)	71 (4.30)	78 (4.12)
Advice, references	6 (2.5)	36 (2.18)	42 (2.22)
Help with a choice	4 (1.6)	19 (1.15)	23 (1.22)
Lifestyle advice	3 (1.2)	30 (1.82)	33 (1.74)
Unknown	2 (0.8)	3 (0.18)	5 (0.26)
Help with essay/paper	0 (0.0%)	47 (32.85)	47 (2.48)

**Table 5:** Topics of required specific information on lung cancer and lung cancer therapy, patients, and other questioners.

Topics of specific information	Patients (n=145) n (%)	Other questioners (n=931) n (%)
Therapy side-effects	29 (20.0)	100 (10.7)
Diagnostics	28 (19.3)	113 (12.1)
Regular therapy	26 (17.9)	196 (21.1)
Experimental therapy	15 (10.3)	50 (5.4)
Disease symptoms	14 (9.7)	109 (11.7)
What can it be?	11 (7.6)	52 (5.6)
Disease course	9 (6.2)	102 (11.0)
General information on lung cancer	7 (4.8)	140 (15.0)
Other	5 (3.4)	51 (5.5)
Alternative therapy	1 (0.7)	6 (0.6)
Lung cancer prevention	0 (0.0)	12 (1.3)

## Discussion

### Principal Findings

In this study, we looked at information that was requested from online lung cancer specialists by visitors of the DLIC website. Most questions were asked by lung cancer patients and their caregivers (especially daughter and granddaughter). There are many studies published about looking for health related information on the Internet, but studies about asking specific questions to online (lung) specialists are rare. This distinguishes our present study. Our study results show that most frequently requested information was more detailed information about lung cancer (such as regular therapy, diagnostics, and disease symptoms), verification of doctor’s words, diagnosis and prognosis. This kind of information could have been obtained from treating physicians, implying that the supplied information to lung cancer patients and their caregivers may be insufficient with regard to their needs. Beside this, the impressive number of questions asked on the website indicates that patients and caregivers are willing to participate in online dialogues with specialists.

Before comparing our results with data from other studies, it should be mentioned that we have chosen to analyse all questions regardless if they were from one person or a different one. Since the number of persons who asked more than 1 question is substantial (20%), this may have influenced our results. An argument for our approach is that each question was different and was asked during different phases of lung cancer procedure. Each question should thus be considered as one item regardless of who asked it.

Similarities and differences between our results and other study results can be seen. We found that (1158/1893, 61%) of the questions were asked by caregivers. The result confirms our observations in our previous study [11] and other studies that a large percentage of caregivers use the Internet. Norum et al [16] reported that 60% of patients’ partners used Internet and Ybarra et al [17] found that support seekers were significantly more likely to be patients’ caregivers.

In our study, (243/1893, 13%) of the questions were asked by patients. Studies of Fleisher et al [18] and Mold et al [19] stated that 15 to 20% of patients in their study were *indirect* Internet users. Miles et al [20] gave a percentage of 24%. Our results are different and add to existing study data because present study gives the percentage of patients who are *direct* Internet users. Furthermore, our study group only included lung cancer patients. According to Eysenbach’s study, only 16% of all information seeking cancer patients was a lung cancer patient [21], which is more comparable to our findings. This relatively low percentage of lung cancer patients looking for information and asking questions online could be explained by differences in gender, age, and socio-economic status. The majority of questioners were young women and the biggest group of questioners was a daughter or granddaughter. Women look for health related information on the Internet more often than men and a younger age is associated with a greater Internet use [3,16,21-30]. It is known that lung cancer patients usually are elderly males of low social levels. This is associated with a limited tendency to use the Internet [31]. Although data on age and gender of all patients who asked questions on the website were not completely available, we assume that the Dutch lung cancer patients do not differ from lung cancer patients elsewhere and thus go on the Internet less often than their female caregivers. Additionally, lung cancer patients’ strategies to look for medical information differ from other cancer patients. They are more likely to be passive in seeking information than other cancer patients [32]. Thus, they will ask their caregivers to look for information for them and look less actively themselves. In this manner, a lot of lung cancer patients were getting information from the DLIC website indirectly.

Considering the number of visitors per year attending the website and the number of questions asked by caregivers and lung cancer patients, the question rises whether present results are representative for the total website visiting population of caregivers and lung cancer patients. According to our data, about 212 questions are being asked by caregivers per 100 patients per year for the 14,000 annual cases in the Netherlands. The number of visitors of the DLIC website per year appears relatively larger than the number of questions asked. A plausible explanation for this fact may be that many visitors already found the answers to their questions in the websites general information or in the questions in the section “Ask the physician”. Another explanation may be that visitors solely visited the website to look for information and that some of them may not dare to ask questions. If we compare present results with the poll “visitor identity” we had performed in our previous study [11], the percentages of lung cancer patients and caregivers correspond well with each other. Thus, questions asked by visitors on the website are quite representative for the total visitor population.

Our study found that most information seeking behavior occurred during therapy, after therapy, and after diagnosis. Other studies show similar results, indicating that most patients seek explanatory information just after their diagnosis and before starting treatment; or just after diagnosis (49%) or during treatment (31%) [33,34].

Information seekers had specific questions. Most of them wanted specialized information about a specific topic concerning lung cancer, a diagnosis, a prognosis, or to verify doctor’s information. The most frequently asked topics of specific information about lung cancer in present study were regular therapy, diagnostics, general information about lung cancer, therapy side effects and disease symptoms. A number of studies investigated the most wanted information topics by Internet users, and found that information related to treatment (80%) [35], information about a condition, symptoms, advice about symptoms and treatment [36], information on cancer screening/diagnosis, support services, psychosocial issues, and general cancer site information [37], were the most wanted topics. Rutten et al [38], found that the most frequent information needs of cancer patients were information on treatment (38.1%), specific on cancer (12.8%), rehabilitation (12.2%), and prognosis (10.8%).

It is noteworthy that patients of our study were more interested in trials and side effects than the other questioners, who were mostly caregivers.

### Further Research

Given the questions on the webpage “ask the Physician” and the fact that most answers could have been obtained from the treating physician/specialist, it could be concluded that for many of these lung cancer patients and caregivers visiting the website, information given during specialist consultations was unclear, insufficient, not well understood or not well remembered. This has also been mentioned in several studies [39-41]. However, since we do not know whether caregivers asking questions on the website were actually present during consultations with treating physicians, we cannot conclude that the given information was indeed unclear and insufficient to patients or to them. Neither can we conclude that the information was not given, not well understood, or not well remembered because we were not present during consultations as well. Submission of a question does not necessarily indicate that information has not been provided. We do not know what information has been given. Beside this, investigators in a recent study have found age and prognosis to be predictive for poor information recall in cancer patients [42]. Patients and caregivers may have had difficulties to remember medical information. Additionally, the information needs of lung cancer patients differ from their caregivers’, as illustrated by our study. Nevertheless, as noticeable in our results, (151/1893, 8%) of the questions concerned an explanation of doctor’s words or terminology, indicating that a (small) part of the information given by treating specialists is actually not clear. Also the large number of questions on the website still is a signal that the medical information supply of lung cancer patients and their caregivers does not completely match their information needs. This phenomenon is an interesting indication that lung cancer patients and their caregivers are open and willing to participate in online dialogues with treating specialists. In our previous study [11], we already showed with a visitor satisfaction poll that the majority of visitors were very positive about the usefulness of the website and its interactive page. Thus, we suggest an adaptation, and hopefully subsequently possible amelioration of the medical information supply to lung cancer patients and their caregivers. Suggestions for improvement could be to survey repeatedly about the information needs of lung cancer patients and their caregivers, giving printed or written information to patients and caregivers [43-45], encouraging email contact and online dialogue with specialists for questions [8,9], directing to reliable Internet sources of information for complementary information [11], and repeat the information given during consultations.

Further research is needed to explore the reasons why lung cancer patients and their caregivers turn to online lung specialists for information. The importance and role of caregivers during treatment should also be investigated since they appear to be involved in the information supply of lung cancer patients in present study.

### Conclusions

Lung cancer patients and their caregivers asked most questions in the interactive section of the DLIC website. The most frequently requested information was more detailed and specific information about lung cancer (regular therapy, diagnostics and disease symptoms), verification of what the doctor has said, diagnosis, and prognosis. Most of the requested information could have been obtained from treating specialists, indicating that the information supply of lung cancer patients and their caregivers may not be matching their needs sufficiently. Since lung cancer patients and caregivers seem to be appreciating and willing to use online interactive dialogue with lung cancer specialists, further implementation of such dialogue might be a solution.

## Abbreviations

DLIC: Dutch Lung Cancer Information Centre
